# Roles of Parental Psychological Flexibility, Self-Compassion, and Self-Efficacy in Affecting Mental Health and Quality of Life in Parents of Children with Eczema

**DOI:** 10.3390/healthcare11202708

**Published:** 2023-10-10

**Authors:** Yuen Yu Chong, Joycelyn Yee Man Kwan, Pui Tik Yau, Ho Yu Cheng, Wai Tong Chien

**Affiliations:** The Nethersole School of Nursing, Faculty of Medicine, The Chinese University of Hong Kong, Hong Kong SAR, China; joycelynkwan@cuhk.edu.hk (J.Y.M.K.); jamyau@cuhk.edu.hk (P.T.Y.); hycheng@cuhk.edu.hk (H.Y.C.); wtchien@cuhk.edu.hk (W.T.C.)

**Keywords:** psychological flexibility, self-compassion, mental health, quality of life, parents, eczema

## Abstract

Parents of young children with eczema often experience adverse mental health consequences, including depression, anxiety, stress, and a reduced health-related quality of life (HRQoL), due to the unpredictable nature of flare-ups and exacerbations. This study investigated the roles of psychological flexibility, self-compassion, and self-efficacy in fostering parental mental health outcomes and HRQoL while caring for children diagnosed with eczema. Baseline data from an ongoing clinical trial examining the effects of a family acceptance-and-commitment-therapy-based eczema management program (FACT-EMP) on parent–child dyads affected by eczema (*N* = 110 dyads, 75.5% mothers; 66.4% boys) were analyzed using adjusted hierarchical regression analyses. The findings indicate that psychological inflexibility was significantly associated with symptoms of anxiety, depression, stress, and HRQoL. Self-compassion was significantly linked to all assessed mental health outcomes, whereas self-efficacy showed a significant association only with symptoms of depression. These results underscore the significance of promoting parental psychological flexibility and self-compassion through acceptance and commitment therapy and compassion-based approaches to enhance mental health and quality of life while managing children’s eczema.

## 1. Introduction

Eczema is a chronic and relapsing inflammatory skin condition characterized by eczematous lesions and intense pruritus [1]. The latest 2023 international study arising from the International Study of Asthma and Allergies in Children (ISAAC) has indicated that at least 6% of children worldwide are suffering from symptoms of current eczema, such as chronic rashes, xerosis, and crusting, with an increase in the lifetime prevalence of at least 2% over the past decade [2]. In Hong Kong, around 30% of Hong Kong children (approximately 160,000 children) have eczema [3]. It is widely recognized that children with eczema generally experience a lower quality of life and worse mental health conditions than healthy individuals due to the severe symptoms of eczema, which cause sleep disturbances such as poor initiation [4,5], frequent awakenings [6,7], and prolonged nocturnal wakefulness [8]. Stigmatization by peers, including being teased or bullied for skin conditions arising from eczema, is also well documented [9].

Considering the complex etiology of eczema, arising from a combination of genetic predisposition and environmental elements, effectively managing and mitigating the condition requires parent–child dyads to engage in frequent medical evaluations and adjustments to treatment [10]. This requires the dyads to adapt their family routines accordingly, thereby introducing additional complexity and placing a significant psychological burden on parents [11,12]. Indeed, the unpredictable nature of eczema flare-ups, characterized by unforeseen and sudden exacerbations of symptoms (e.g., aggravated skin lesions) in different body locations without identifiable triggers, coupled with inconsistent treatment responses, demands constant vigilance from parents and the adoption of various preventive strategies [13]. These strategies include identifying and avoiding triggers, implementing a consistent skincare routine, and ensuring regular and thorough moisturization [14]. Extant evidence indicates a positive association between the severity of eczema symptoms in children and adverse mental health consequences in their parents, such as symptoms of depression and anxiety [15,16], feelings of helplessness [17], and heightened levels of worry, exhaustion, and stress [18,19]. Given the time-consuming and exhausting nature of caring for children with eczema and the resulting adverse mental health outcomes, the quality of life of parents is often immensely impacted [20].

In the context of managing a child diagnosed with eczema, parental self-efficacy has been found to play a crucial role in affecting the mental health and health-related quality of life (HRQoL) of parents. According to Bandura’s social cognitive theory, self-confidence is linked to the concept of self-efficacy, which pertains to individuals’ beliefs in their abilities to positively influence their health by effectively managing their behavior [21]. This theory implies that for a parent or caregiver, with a higher level of self-efficacy, a greater ability to monitor eczema symptoms and administer eczema treatments for their children would be likely. It is well documented that parents who possess high levels of self-efficacy acquire great perceived confidence in managing their child’s health conditions, thereby resulting in better mental health outcomes and HRQoL [22,23].

### 1.1. Psychological Flexibility and Self-Compassion

A growing body of evidence has examined the potential protective roles of accepting internal experiences in enhancing the psychological adjustment of parents while navigating the illness trajectory of their children with chronic conditions [24]. This emphasis on acceptance has led to the investigation of two therapeutic processes, namely, psychological flexibility and self-compassion, both sharing the common feature of shifting focus away from the specific contents of internal experiences to a person’s relationship with these experiences [25]. Indeed, recent studies conducted in general population samples [26], patients with chronic pain [27], and parents [28] have advocated the need to study these two therapeutic constructs together to examine their impacts on mental health and quality of life. Psychological flexibility refers to an individual’s capacity to remain connected to the present moment, engage in an open-and-aware cognitive process, and take actions in accordance with their personally held values [29]. Empirical evidence has suggested that individuals who are more psychologically flexible report lower levels of stress, depression [30], anxiety [31], and insomnia [32,33,34], as well as better overall well-being [35,36,37]. In the context of caregiving, parental psychological flexibility refers to the acquired ability of parents to effectively adapt and respond to the challenging psychological experiences and demands associated with parenting while simultaneously adhering to appropriate parenting practices that align with their personal values [38]. An increasing number of studies have supported the role of parental psychological flexibility in fostering better mental health outcomes and HRQoL of parents, as well as other health outcomes of their children diagnosed with various chronic conditions, such as autism spectrum disorders [39,40], acquired brain injuries [41], and cerebral palsy [42]. Self-compassion refers to the practice of extending kindness to oneself, recognizing the universality of the human experience, and mindfully acknowledging adverse thoughts and emotions without excessive attachment [43]. Review evidence has shown a significant and negative association between self-compassion and a range of mental health symptoms, including depression [44], anxiety [44], and stress [45]. Self-compassion can also be conceptualized as a set of skills that can be cultivated and strengthened through practical exercises [46]. Parents who possess high self-compassion tend to be more aware of their emotional experiences and warmth, prioritize self-care with kindness and warmth, recognize their difficulties as shared experiences, and consequently protect themselves from poor psychological outcomes [47].

### 1.2. The Present Study

Both theoretical frameworks and empirical studies consistently highlight the pivotal roles of psychological flexibility and self-compassion in improving the mental health and HRQoL of caregivers dealing with chronic conditions in their children. This body of research underscores the positive impacts of these psychological constructs, ranging from reduced psychological distress to enhanced overall well-being, across various caregiving contexts. However, these roles remain unexplored in families with children diagnosed with eczema. The existing literature is predominantly focused on parental self-efficacy [23,48], leaving a critical gap in our understanding of the unique psychological aspects experienced by parents of children with eczema. Recognizing these relationships is essential for developing eczema management programs that address the distinct psychological challenges encountered by these parents. Additionally, studying mental health symptoms, including anxiety, depression, and stress, alongside HRQoL in this population serves two key purposes. Firstly, it aims to uncover the potential protective roles of psychological flexibility and self-compassion in mitigating the adverse psychological effects commonly associated with chronic caregiving. Secondly, it provides a comprehensive understanding of the overall well-being of these parents, offering valuable insights into their challenges and the factors that can alleviate or exacerbate these challenges. In this study, we aimed to examine whether the two therapeutic processes, namely, psychological flexibility and self-compassion, together with eczema management self-efficacy, were associated with mental health symptoms (anxiety, depression, and stress) and HRQoL in a sample of Hong Kong Chinese parents of young children diagnosed with eczema. Our hypothesis posits that psychological flexibility, self-compassion, and eczema management self-efficacy will all be simultaneously and positively associated with HRQoL and mental health symptoms among parents caring for their children with eczema.

## 2. Materials and Methods

### 2.1. Study Procedures

Employing a cross-sectional design, this study utilized baseline assessment data from an ongoing randomized controlled trial investigating the effects of a family acceptance-and-commitment-therapy-based eczema management program (FACT-EMP) on the health outcomes of parent–child dyads affected by eczema, as preregistered at ClinicalTrials.gov (NCT04919330). Between July 2021 and December 2022, participants were consecutively recruited from three pediatric outpatient clinics under the Department of Paediatric and Adolescent Medicine of a regional hospital serving three districts in Hong Kong; these districts serve 16.3% of the Hong Kong population of children under the age of 14 years [49]. All parent–child dyads who attended the outpatient clinics for follow-up assessments and treatments related to eczema care were screened by a trained research assistant to assess eligibility. Written informed consent was obtained from the participants before the screening assessment and the completion of the baseline assessment by using a self-administered questionnaire.

### 2.2. Participants

Parents were eligible to participate if they were aged 19 or above; self-identified as the primary caregiver of a child aged 6 to 12 years who was diagnosed with eczema by a physician, as documented in the electronic medical records based on the International Classification Diseases-10 codes L20–L30; living together with the target child; able to communicate in Cantonese; and accessible by telephone. Parents were excluded from the study if they had already participated in another intervention study related to pediatric eczema management, if they presented with clinically significant psychiatric morbidity, and/or if their child had significant medical and/or developmental comorbidities. The data were collected during the midst of the COVID-19 pandemic, during which various quarantine and social distancing measures were implemented; hence, the attendance of the study clinics only reached up to 40% of the usual, so it took us more time to complete the data collection. Throughout the study period, over 2.6 million people (36% of the total population) in Hong Kong were infected with COVID-19. Governmental statistical data further revealed that a substantial proportion, exceeding 60%, of children within the population also contracted the virus [50]. Ethical approval was obtained from the study hospital and the university before the study commencement.

### 2.3. Measures 

Psychological inflexibility. The 7-item Acceptance and Action Questionnaire-II (AAQ-II) was employed to assess psychological inflexibility. Parents were asked to indicate their agreement with a series of statements, such as ‘My painful experiences and memories make it difficult for me to live a life that I would value’, on a 7-point Likert scale ranging from 1 (never true) to 7 (always true) [51]. Higher total scores indicate more psychological inflexibility. The AAQ-II has demonstrated strong internal consistencies (α = 0.86 to 0.88) and test–retest reliabilities in Chinese population groups (*r*  =  0.74 to 0.86) [52] (Cronbach’s α in this study = 0.88).

Self-compassion. The 26-item Self-Compassion Scale (SCS) was used to assess how often the parents respond to difficult situations about eczema care for their children with self-kindness, self-judgment, common humanity, isolation, mindfulness, and over-identification [53]. An example statement was ‘When I’m going through a very hard time, I give myself the caring and tenderness I need’. Response options on a 5-point Likert scale ranged from 1 (Almost Never) to 5 (Almost Always), with a higher total score indicating greater self-compassion [53]. The SCS demonstrated good internal consistencies in both Chinese (α = 0.84) [54] and Western population groups (α = 0.66 to 0.91) [55,56,57] (Cronbach’s α in this study = 0.85 for the overall SCS, 0.76 to 0.83 for the SCS subscales) [58].

Eczema management self-efficacy. The 29-item Parental Self-Efficacy with Eczema Care Index (PASECI) was used to assess the self-efficacy of parents in managing their child’s eczema, including performing management tasks and managing symptoms [23]. Parents were asked to indicate the degree of perceived competence in completing a range of tasks related to eczema care arising from the following four domains: managing medication (e.g., ‘correctly use steroid creams for your child’), managing eczema and symptoms (e.g., ‘manage to avoid things that irritate/aggravate your child’s eczema’), communicating with healthcare professionals (e.g., ‘tell the GP when your child’s eczema is not getting better’), and managing personal challenges (e.g., ‘without support from my family or friends’). Response options on an 11-point Likert scale ranged from 0 (cannot do at all) to 10 (highly certain can do it). A higher total score indicates greater parental self-efficacy. The PASECI exhibited high internal consistency (α =  0.97), test–retest reliabilities (ICC = 0.93–0.99), and acceptable convergent validity in both Western and Asian population groups (Cronbach’s α in this study = 0.82 for the overall PASECI, 0.82 to 0.91 for its subscales) [59].

Mental health symptoms. The 21-item Depression Anxiety Stress Scale 21 (DASS-21), a shortened version of the original DASS-42, was utilized to evaluate the symptoms of depression (e.g., ‘I felt that I had nothing to look forward to’), anxiety (e.g., ‘I felt I was close to panic’) and stress (e.g., ‘I found myself getting agitated’) [60]. Parents were asked to indicate the extent to which each statement reflected their experiences in the past week using a 4-point Likert scale, ranging from 0 (does not apply to me at all) to 3 (applies to me very much, or most of the time). Higher scores on each subscale suggest more severe symptoms. The DASS-21 demonstrated good reliabilities of 0.82, 0.88, and 0.90 for the depression, anxiety, and stress subscales [60]. The Chinese version of DASS-21 has also been validated among various Chinese population groups [61] (Cronbach’s α in this study = 0.87 for the overall DASS-21, 0.79 to 0.87 for its subscales).

Health-related quality of life. The 28-item Parents’ Index of Quality of Life in Atopic Dermatitis (PIQoL-AD) was employed to assess the six practical aspects of the HRQoL of parents in caring for their children diagnosed with eczema: sleep, daily activities, emotional well-being, family relationships, financial impact, and treatment issues. Parents rated 28 statements in a dichotomous yes/no response format with 0 (No) and 1 (Yes) (e.g., ‘Looking after him takes a lot of effort’). A high score indicates poor HRQoL. The PIQoL-AD is a standardized measure that possesses good internal consistency (α = 0.88) and test–retest reliability (*r* = 0.85) in various population groups [62] (Cronbach’s α in this study = 0.85).

Sociodemographic characteristics. The sociodemographic variables of parents were assessed, including age, gender, marital status, level of education, monthly household income, and occupational and family status. Parents were asked to report their child’s demographics and eczema conditions in the self-administered questionnaire, including age, gender, age of eczema onset, family history of atopy, and healthcare service utilization related to the flare-up of eczema symptoms. Notably, the child’s eczema symptoms were assessed via a parental proxy report using the Patient-Oriented Eczema Measure (POEM) [63]. The 7-item POEM is a brief questionnaire inquiring about the number of days that the child experienced eczema-related symptoms in the past week using a 5-point Likert scale, ranging from 0 (no days) to 4 (every day). A higher score indicates more severe symptoms. The POEM is a 7-item questionnaire inquiring about eczema-specific symptoms in the preceding week; each item is scored 0–4, for a total score of 0–28 [63]. A decrease in the POEM score indicates a reduction (improvement) in the frequency of symptoms of eczema. The POEM score is one of the key patient-reported outcomes (PROs) in eczema-related clinical trials [64].

### 2.4. Data Analyses 

The normality of continuous variables was assessed by examining skewness statistics and normal probability plots. Following the suggestion by Muthen and Kaplan [65], skewness and kurtosis values within the range of −1 to +1 were considered acceptable indicators of normality. The sociodemographic and clinical characteristics of parents and their children diagnosed with eczema are presented using descriptive statistics, including means, standard deviations, and frequencies. Our study aimed to investigate the impact of three independent variables, namely, psychological inflexibility, self-compassion, and self-efficacy, on specific parental outcomes, including symptoms of anxiety, depression, stress, and HRQoL. We employed hierarchical regression models for our analysis. In the initial block of these models, we incorporated covariates that had shown significant associations with the dependent variables. Subsequently, in the second block, we introduced psychological flexibility, self-compassion, and eczema management self-efficacy as predictors in the second block. To identify appropriate covariates for inclusion in the regression models, we conducted a series of statistical tests. For continuous potential covariates, we performed Pearson correlation analysis to evaluate their relationships with the dependent variables. For potential bivariate covariates, independent-sample t-tests were employed to assess their associations with the dependent variables. Furthermore, for potential categorical covariates with three or more levels, we used ANOVA tests to examine their relationships with the dependent variables. Variables that exhibited significant associations with each parental outcome were included as covariates in the first block of two-block hierarchical regression models. To assess multicollinearity, we examined variance inflation factor statistics. All statistical analyses were performed using IBM SPSS version 26.0 (IBM Crop., Armonk, NY, USA). All statistical tests were two-sided, and the significance level was set at 0.05.

## 3. Results

### 3.1. Characteristics of the Participants

Table 1 displays the sociodemographic and clinical characteristics of the parents and their children diagnosed with eczema (*N* = 110). The Kolmogorov–Smirnov test (*P*s ranged from 0.200 to 0.241) and Shapiro–Wilk test (*P*s ranged from 0.106 to 0.119) for parental outcomes were non-significant. The skewness and kurtosis values of these parental outcome variables fell within the range of −0.30 to 0.87, indicating the acceptable normality of the data. The mean (SD) age of the parents was 41.9 (8.3) years, with most being mothers (75.5%) and full-time housewives (50.0%). Moreover, over 90% of the parents had obtained at least a secondary level of school education. The mean (SD) age of the children was 8.2 (2.4) years, and 66.4% of them were boys. The sampled children were diagnosed with eczema at a mean age of 23.1 months. The mean (SD) score of the POEM was 11.0 (7.3), with half of the children experiencing itchiness every day and over 15% having their sleep affected by eczema symptoms and experiencing scratching until bleeding. Additionally, over one-fifth of the sampled children had attended unscheduled general outpatient visits due to eczema exacerbations over the past 12 months, while less than 4% required hospitalization. The possible range, mean (SD), and score distribution for each parental measure are displayed in Table A1, indicating that nearly 20% of the parents experienced at least mild levels of depression, anxiety, and stress symptoms.

### 3.2. Univariate Analyses and Hierarchical Regression Analyses

The univariate association analyses (i.e., correlation analyses, independent t-tests, and ANOVAs) between the sociodemographic and clinical characteristics of parent–child dyads and parental outcomes (i.e., HRQoL and mental health symptoms) are shown in Table A2. Educational level (*P* = 0.035 for the PIQoL-AD total score), family status (*P* = 0.032 for the PIQoL-AD total score; *P* = 0.034 for the DASS-21 Anxiety score), family history of atopy (*P* = 0.004 for the DASS-21 Anxiety score; *P* = 0.004 for the DASS-21 total score), and eczema severity indicated by the POEM score (*r* = 0.37, *P* ≤ 0.001 for the PIQoL-AD total score; *r* = 0.19, *P* = 0.042 for the DASS-21 Anxiety score) were found to be significantly associated with the corresponding parental outcomes, and they serve as the covariates in the hierarchical regression models.

Table 2 presents the results of the hierarchical regression analyses examining the roles of parental psychological flexibility, self-compassion, and self-efficacy in affecting mental health and quality of life. The models examining HRQoL (χ^2^ (7) = 14.47, *P* < 0.001), symptoms of depression (χ^2^ (3) = 35.10, *P* < 0.001), anxiety (χ^2^ (6) = 14.01, *P* < 0.001), stress (χ^2^ (3) = 30.55, *P* < 0.001), and overall mental health symptoms (χ^2^ (4) = 29.36, *P* < 0.001) yielded statistically significant results. In the model assessing HRQoL as the outcome, parental education level, family status, POEM score, and general outpatient visits accounted for 17% of the variance in Step 1. In Step 2, psychological inflexibility, as measured by the AAQ-II (standardized beta coefficient, β, = 0.62, *P* < 0.001), significantly improved the model, contributing an additional 34% of the variance increase (*P* < 0.001). The final model explained 51% of the variance. For the model assessing symptoms of depression, psychological inflexibility (β = 0.35, *P* < 0.001), self-compassion (β = −0.39, *P* < 0.001), and eczema management self-efficacy (β = −0.16, *P* = 0.029) collectively accounted for 50% of the variance in the regression model. Psychological inflexibility (β ranged from 0.25 to 0.39, *P* ranged from <0.001 to 0.008) and self-compassion (β ranged from −0.39 to −0.38, all *P*s ≤ 0.001) remained significant factors in the models assessing symptoms of anxiety, stress, and overall mental health symptoms, explaining at least 35% of the variance increase (all *P*s ≤ 0.001). The final models accounted for total variances ranging from 45% to 53%.

## 4. Discussion

The present study investigated the roles of psychological flexibility, self-compassion, and eczema management self-efficacy on HRQoL and mental health symptoms among a sample of parents of children diagnosed with eczema in Hong Kong. Our hypotheses were partially supported, as indicated by the results of adjusted hierarchical regression analyses. When all three factors (psychological flexibility, self-compassion, and eczema management self-efficacy) were included as simultaneous predictors, psychological flexibility emerged as the only factor associated with all parental outcomes, including HRQoL and symptoms of depression, anxiety, and stress. Self-compassion showed associations with all measured mental health symptoms but did not significantly relate to HRQoL. On the other hand, eczema management self-efficacy was associated solely with symptoms of depression and did not show significant associations with other parental outcomes.

The significant impact of parental psychological flexibility, as measured by the level of inflexibility using the AAQ-II scale, on both HRQoL and the mental health outcomes of parents caring for children with eczema aligns with findings from prior studies. These studies consistently demonstrate that parents who exhibit greater psychological flexibility tend to have better mental health and enhanced caregiving capabilities when caring for their children experiencing various types of medical health conditions, including asthma [66], chronic pain with significant pain-related disabilities [67], and cancer or life-saving cardiac surgeries [68]. Notably, our research team has been actively investigating the potential role of parental psychological flexibility since 2017, particularly in relation to the mental health of parents and the asthma symptoms of their children [66,69]. This was further supported by our 2019 clinical trial that focused on an acceptance-and-commitment-therapy-based pediatric asthma management program for parents, which aimed to foster psychological flexibility in parents for improving their mental health and asthma care [70]. Building upon these previous findings, the present study expands the current understanding by demonstrating the positive influence of parental psychological flexibility in families of children diagnosed with eczema. Importantly, it should be acknowledged that there are plausible biological pathways that have been elucidated, highlighting eczema as the initial stage in an atopic march, which can ultimately progress to asthma [71]. This is also supported by the shared immune dysregulation and inflammatory mechanisms observed in both conditions [72]. Considering the similarities between asthma and eczema, the present study expands our understanding that parental psychological flexibility is valuable not only for families of children with disabilities but also for those affected by other common chronic inflammatory conditions in children, such as asthma, eczema, and chronic pain. It has a significant impact on parents’ mental health and overall quality of life and also influences the well-being of their children.

Similar to psychological flexibility, parents with high levels of self-compassion also demonstrated lower levels of symptoms related to depression, anxiety, and stress. This finding aligns with existing research that consistently supports the beneficial role of self-compassion in parental mental health [47]. Studies focusing on parental caregiving consistently indicate that parents who cultivate higher levels of self-compassion experience reduced levels of parenting stress, guilt, shame, and self-criticism [73] while concurrently reporting greater levels of parenting satisfaction, competence, and overall well-being [47,73]. Our study further contributes to this understanding by highlighting the significance of self-compassion in the mental health of parents of children with eczema. This suggests that, alongside psychological flexibility, self-compassion may serve as another valuable resource for parents facing challenges and stressors associated with raising children with chronic inflammatory conditions. Self-compassion, characterized by emotional awareness and self-kindness during difficult times (e.g., a child’s emotional outburst during eczema exacerbations requiring immediate medical attention), primarily focuses on promoting emotional well-being and reducing self-criticism [45]. In contrast, psychological flexibility, encompassing acceptance, present-moment awareness, and adaptive coping strategies and behaviors, enables parents to manage practical aspects of caregiving (e.g., managing childhood eczema symptoms), engage in problem solving, and persevere through difficulties [36]. Hence, our findings reveal that parental psychological flexibility exerts a comprehensive influence on both the mental health outcomes and objective indicators of quality of life concerning childhood eczema care, while self-compassion primarily contributes to parents’ mental health outcomes.

The non-significant effect of self-efficacy on the mental health and HRQoL of parents in the context of caring for children with eczema, when included as a predictor alongside psychological flexibility and self-compassion, may be attributed to several plausible reasons. Firstly, psychological flexibility and self-compassion encompass aspects related to coping strategies, emotional well-being, and adaptive responses, which may overlap with the concept of self-efficacy to some extent [74,75]. Consequently, including self-efficacy alongside these constructs may result in redundancy, reducing its unique contribution to the measured outcomes. Secondly, it is plausible that self-efficacy operates through mediating or moderating pathways rather than having a direct impact on mental health and HRQoL outcomes. For instance, parents with high self-efficacy in pediatric eczema care may perceive failures (e.g., experimenting with different treatment approaches for their child but failing to yield the desired results) as opportunities for growth, fostering self-forgiveness and forgiveness toward others. This mindset aligns with the core components of self-compassion and may lead to improved mental health outcomes and HRQoL. Simultaneously, self-efficacy facilitates more psychological flexibility in parents by encouraging them to explore new coping strategies, seek assistance, and avoid rigid patterns in eczema care. These processes of psychological flexibility, in turn, may contribute to enhanced well-being and quality of life for parents. It is important to acknowledge that self-efficacy does not operate in isolation [76] but potentially interacts with other factors, such as psychological flexibility and self-compassion. Exploring the interplay of these constructs in the context of pediatric eczema management is an area worthy of future investigation.

Several limitations of this study should be acknowledged. Firstly, the study was conducted at a single center, specifically a regional hospital in Hong Kong, during the challenging period of the COVID-19 pandemic. This reduced the generalizability of the findings, as the severity of the children’s eczema symptoms and the mental health symptoms of their parents could have been influenced by the pandemic. However, it is worth noting that the sociodemographic characteristics of the sampled children in our study, such as the gender distribution (over 60% were boys), age of eczema diagnosis (at the age of 2 years), and proportion of children affected by eczema symptoms (over 15% experienced chronic symptoms, such as difficulty sleeping), were similar to those observed in a recent local population-based ISSAC study that examined the prevalence of allergic diseases among primary and secondary schoolchildren in Hong Kong [3]. Secondly, this study used AAQ-II to assess psychological inflexibility, in which higher scores indicate that the person is more psychologically inflexible. While the AAQ-II is widely employed to assess psychological flexibility, it has been recently critiqued for its inability to adequately differentiate between responses to experiences and the experiences themselves [29]. This confounding factor may artificially inflate the correlations between the AAQ-II and measures of psychological distress (i.e., DASS-21, as used in this study), as it fails to capture the context in which flexibility is most relevant—the pursuit of valued goals [77]. For future studies, the use of an analysis of subscales (if present) within alternative measures, such as the Personalized Psychological Flexibility Index (PPFI) [78], the Comprehensive Assessment of Acceptance and Commitment Therapy Processes Short-Form (CompACT-10) [79], and the Psy-Flex [80], may allow for a more in-depth analysis of the six processes of psychological flexibility on the parental outcomes. Thirdly, the sample size of fathers (*n* = 17, 15% of sampled parents) in the study was relatively small. This phenomenon is commonly seen in studies of children with eczema [81,82,83], as mothers are often more involved in children’s affairs than fathers, particularly in Hong Kong, where traditional gender roles designate mothers as primary caregivers [84]. However, this imbalance poses limitations, impairing the ability to conduct a subgroup analysis to evaluate potential differences between fathers and mothers regarding psychological flexibility and self-compassion. Future studies should therefore strive to include a larger sample of fathers to further explore the generalizability of the study findings to fathers. Fourthly, it is noteworthy that less than 4% of the children in our study required emergency medical attention and hospitalization due to severe eczema exacerbations. This suggests that our findings may be applicable to families with children experiencing mild levels of eczema symptoms. To enhance the generalizability of our results, future studies could consider expanding recruitment settings from outpatient clinics to inpatient settings, such as pediatric wards, where more severe cases are often hospitalized. Finally, the cross-sectional design of the study did not allow the investigation of the causal relationships between the study variables or constructs. Future research should employ longitudinal designs with follow-up periods. This extended timeframe would not only enable the examination of the causal relationships between these variables but also facilitate mediation analyses to explore how self-efficacy, psychological flexibility, and self-compassion may individually or collectively mediate the relationships between each other and our targeted outcomes. Additionally, considering the impact of seasonal variations on children’s eczema symptoms, as measured by the POEM score, could provide a more comprehensive understanding of the dynamics at play. Such analyses would contribute significantly to uncovering the underlying mechanisms and pathways through which these psychological factors influence parental well-being in the context of caring for children with eczema.

## 5. Study Implications and Conclusions

Parents caring for children diagnosed with eczema often experience poor mental health outcomes and a diminished quality of life, particularly during eczema exacerbations, necessitating consistent skincare routines and trigger avoidance. These findings have crucial implications for clinical practice and research, emphasizing the need to understand, assess, and leverage potential adaptive mechanisms like self-compassion and psychological flexibility to enhance the psychological well-being and quality of life of these parents. In clinical practice, while medical assessments traditionally concentrate on visually evaluating the child’s skin condition, our results stress the importance of evaluating eczema’s impact on family caregivers, including their quality of life and mental well-being. Recognizing signs of caregiver distress and providing appropriate support should be an integral component of healthcare providers’ training for pediatric eczema care. From a research perspective, our study underscores the significance of addressing caregiver well-being by targeting two malleable factors: psychological flexibility and self-compassion. These factors share common prerequisites, such as fostering self-compassion and developing perspective-taking skills. An expanding body of evidence demonstrates the potential advantages of integrating self-compassion into acceptance and commitment therapy (ACT) or combining ACT with compassion-focused therapy [85]. Building upon these insights, future investigations should explore the feasibility and preliminary effectiveness of merging ACT, which focuses on psychological flexibility, with interventions promoting self-compassion in improving the health outcomes of both parents and their children with eczema. In conclusion, this study sheds light on the intricate relationship between parental psychological flexibility, self-compassion, and the mental health and quality of life outcomes of parents caring for children with eczema. Our findings underscore the need for a holistic approach in clinical practice, recognizing the substantial impact of eczema on family caregivers.

## Figures and Tables

**Table 1:** Characteristics of the parents and their children diagnosed with eczema.

Variables	*n* (%)
Characteristics of parents	
Age (years), mean (SD)	41.9 (8.32)
Relationship with child	
Father	17 (15.5)
Mother	83 (75.5)
Grandparents	10 (9.0)
Marital status	
Single/divorced/widowed	16 (14.5)
Married	94 (85.5)
Education attainment ^1^	
Primary education or below	8 (7.3)
Secondary education	61 (57.0)
Tertiary education or above	38 (35.5)
Monthly household income (HKD) ^1^	
Comprehensive Social Security Assistance	3 (2.7)
<10,000	10 (9.1)
10,000–40,000	64 (58.2)
40,001–60,000	18 (16.4)
>60,000	12 (10.9)
Occupation ^1^	
Housewife	56 (50.9)
Manager	7 (6.4)
Professional	9 (8.2)
Clerk	11 (10.0)
Service and Sales	5 (4.5)
Others ^2^	16 (14.5)
Unemployed	2 (1.8)
Family status	
Nuclear family	68 (61.8)
Single family	8 (7.3)
Extended family	30 (27.3)
Others	4 (3.6)
Personal history of atopy ^3^	
Yes	58 (52.7)
No	52 (47.3)
Family history of atopy ^3^	
Yes	75 (68.2)
No	35 (31.8)
Characteristics of children	
Age (years), mean (SD)	8.23 (2.42)
Gender	
Male	73 (66.4)
Female	37 (33.6)
Age at eczema onset (months), mean (SD)	23.13 (20.2)
Eczema severity by POEM, mean (SD), possible range = 0–28	11.0 (7.3)
Unscheduled general outpatient visits due to eczema exacerbations over the past 12 months	
None	83 (75.5)
1–2 visits	19 (17.3)
3–4 visits	4 (3.6)
≥5 visits	4 (3.6)
Unscheduled private practitioner’s clinic visits due to eczema exacerbations over the past 12 months	
None	87 (79.1)
1–2 visits	14 (12.7)
3–4 visits	2 (1.8)
≥5 visits	7 (6.4)
Emergency care visits due to eczema exacerbations over the past 12 months	
None	106 (96.4)
1 visit	4 (3.6)
Hospitalization due to eczema exacerbations over the past 12 months	
None	106 (96.4)
1 visit	4 (3.6)

Note. *n* = total number of subjects; HKD = Hong Kong Dollars (1 USD = 7.8 HKD); POEM = Patient-Oriented Eczema Measure. ^1^ Missing values < 5%. ^2^ Other occupations include security guards, civil servants, nurses, clinic assistants, and freelancers. ^3^ History of atopy encompasses conditions including asthma, allergic rhinitis, hay fever, or food allergies.

**Table 2:** Hierarchical regression analyses examining the roles of parental psychological flexibility, self-compassion, and self-efficacy in affecting mental health and quality of life.

	PIQoL-AD Total Score ^1^				DASS-21 Depression				DASS-21 Anxiety ^2^				DASS-21 Stress				DASS-21 Total Score ^3^			
	B	SE	β	*p*	B	SE	β	*p*	B	SE	β	*p*	B	SE	β	*p*	B	SE	β	*p*
Constant	9.01	2.99	-	0.003	12.50	2.42	-	<0.001	7.52	2.26	-	0.001	14.07	3.19	-	<0.001	33.75	6.97	-	<0.001
Block 1	-	-	-	-	-	-	-	-	-	-	-	-	-	-	-	-	-	-	-	-
	R² = 0.17				-				R² = 0.10				-				R² = 0.03			
Block 2 ^4^																				
AAQ-II	0.36	0.06	0.62	<0.001	0.13	0.03	0.35	<0.001	0.06	0.02	0.25	0.008	0.17	0.04	0.37	<0.001	0.41	0.09	0.39	<0.001
SCS	0.11	0.17	0.06	0.525	−0.43	0.10	−0.39	<0.001	−0.12	0.02	−0.38	<0.001	−0.55	0.13	−0.39	<0.001	−1.25	0.28	−0.38	<0.001
PASECI	−0.02	0.01	−0.11	0.168	−0.01	0.01	−0.16	0.029	−0.01	0.06	−0.13	0.106	−0.01	0.01	−0.04	0.615	−0.03	0.02	−0.11	0.122
	Total R² = 0.51				Total R² = 0.50				Total R² = 0.45				Total R² = 0.46				Total R² = 0.53			
	(ΔR² = 0.34, *p* < 0.001)				-				(ΔR² = 0.35, *p* < 0.001)				-				(ΔR² = 0.50, *p* < 0.001)			

Note. PIQoL-AD = Parent’s Index of Quality of Life in Atopic Dermatitis; DASS-21 = Depression Anxiety Stress Scale-21; SCS = Self-Compassion Scale; PASECI = Parental Self-Efficacy with Eczema Care Index; AAQ-II = Acceptance and Action Questionnaire-II; B = unstandardized beta coefficients; SE = standard error; β = standardized beta coefficients; *p* = *p*-value; ΔR² = R-Square change. ^1^ For PIQoL-AD total score, parental education level, family status, POEM score, and general outpatient visits were included in Block 1. ^2^ For DASS-21 Anxiety, family status, family history of atopy, and POEM score were included in Block 1. ^3^ For DASS-21 total score, family history of atopy was included in Block 1. ^4^ Stepwise selection of the predictors that were significantly associated with quality of life and depression, anxiety, and stress levels of parents.

## Data Availability

The study reported in this article was not formally preregistered. Neither the data nor the materials have been made available in a permanent third-party archive; requests for the data or material can be sent via email to the lead author at conniechong@cuhk.edu.hk.

## Appendix A

**Table A1:** Parent measures.

	Possible Range	Mean (SD)	*n* (%)
Psychological inflexibility (AAQ-II)			
Total score	7–49	16.2 (9.48)	-
Self-compassion (SCS)			
Total score	6–30	21.0 (3.06)	-
Self-kindness	1–5	3.17 (0.86)	-
Self-judgment	1–5	3.85 (0.78)	-
Common humanity	1–5	3.21 (0.95)	-
Isolation	1–5	3.76 (0.82)	-
Mindfulness	1–5	3.40 (0.95)	-
Over-identification	1–5	3.58 (0.89)	-
Eczema management self-efficacy (PASECI)			
Total score	0–290	217.70 (40.0)	-
Managing medication	0–60	44.45 (11.03)	-
Managing eczema and symptoms	0–60	43.87 (9.71)	-
Communicating with healthcare professionals	0–70	55.80 (11.48)	-
Managing personal challenges	0–100	73.57 (17.71)	-
Mental health symptoms (DASS-21)			
Total score	0–63	9.43 (8.09)	-
Depression	0–21	2.51 (3.42)	-
Normal, *n* (%)	0–4	-	86 (78.2)
Mild, *n* (%)	5–6	-	13 (11.8)
Moderate, *n* (%)	7–10	-	8 (7.3)
Severe, *n* (%)	11–13	-	1 (0.9)
Extremely severe, *n* (%)	≥14	-	2 (1.8)
Anxiety	0–21	2.56 (3.00)	
Normal, *n* (%)	0–3	-	89 (80.9)
Mild, *n* (%)	4–5	-	3 (2.7)
Moderate, *n* (%)	6–7	-	7 (6.4)
Severe, *n* (%)	8–9	-	7 (6.4)
Extremely severe, *n* (%)	≥10	-	4 (3.6)
Stress	0–21	4.35 (4.37)	
Normal, *n* (%)	0–7	-	88 (80.0)
Mild, *n* (%)	8–9	-	10 (9.1)
Moderate, *n* (%)	10–12	-	4 (3.6)
Severe, *n* (%)	13–16	-	7 (6.4)
Extremely severe, *n* (%)	≥17	-	1 (0.9)
Quality of life (PIQoL-AD)			
Total score	0–28	6.99 (5.53)	

Note. AAQ-II = Acceptance and Action Questionnaire-II; DASS-21 = Depression Anxiety Stress Scale-21; *n* = number of participants; PASECI = Parental Self-Efficacy with Eczema Care Index; PIQoL-AD = Parent’s Index of Quality of Life in Atopic Dermatitis; SCS = Self-Compassion Scale.

**Table A2:** Univariate association analyses between characteristics of the parent–child dyads and parental measures.

Characteristics	PIQoL-AD Total Score		DASS-21 Depression		DASS-21 Anxiety		DASS-21 Stress		DASS-21 Total Score	
	Mean (*SD*)/*r*	*p*	Mean (*SD*)/*r*	*p*	Mean (*SD*)/*r*	*p*	Mean (*SD*)/*r*	*p*	Mean (*SD*)/*r*	*p*
Parents’ characteristics										
Age (years)	0.023	0.742	−0.034	0.647	−0.051	0.489	−0.039	0.585	−0.046	0.510
Relationship with child		0.492		0.609		0.848		0.384		0.543
Father	6.2(4.8)		2.2 (3.1)		2.5 (2.3)		3.6 (3.7)		8.3 (8.5)	
Mother	7.3 (5.8)		2.7 (3.5)		2.7 (3.2)		4.6 (4.5)		9.9 (10.5)	
Marital Status		0.280		0.159		0.370		0.608		0.334
Single/divorced/widowed	8.7 (6.9)		3.6 (5.2)		3.2 (3.7)		4.9 (5.1)		11.7(13.1)	
Married	6.8 (5.3)		2.3 (3.0)		2.5 (2.9)		4.3 (4.3)		9.0 (9.5)	
Education attainment ^1^		0.035		0.445		0.448		0.096		0.221
Primary education or below	11.6 (5.0)		3.9 (3.4)		3.6 (3.8)		7.0 (4.5)		14.6 (11.6)	
Secondary education	6.3 (5.1)		2.3 (3.1)		2.3 (3.0)		3.7 (4.0)		8.3(9.4)	
Tertiary education or above	7.4 (6.0)		2.7 (4.0)		2.7 (2.9)		4.9 (4.8)		10.2 (10.8)	
Monthly household income (HKD) ^1^		0.603		0.250		0.994		0.731		0.732
Comprehensive Social Security Assistance	12.0 (6.2)		6.7 (11.5)		3.3 (4.0)		6.7 (8.1)		16.7 (23.7)	
<10,000	7.5 (6.7)		1.7 (3.5)		2.5 (4.3)		3.1 (5.4)		7.3 (12.6)	
10,000–40,000	7.0 (5.7)		2.6 (2.7)		2.6 (2.8)		4.3 (3.9)		9.4 (8.8)	
40,001–60,000	6.3 (5.0)		2.6 (4.2)		2.6 (3.8)		5.0 (5.7)		10.2 (13.2)	
>60,000	6.9 (5.1)		1.8(2.2)		2.4 (1.3)		4.8 (3.2)		9.0 (5.9)	
Occupation ^1^		0.778		0.718		0.708		0.733		0.739
Housewife	7.8 (6.3)		2.8 (3.7)		2.6 (3.4)		4.4 (4.6)		9.8 (10.8)	
Manager	5.6 (3.8)		0.9 (1.1)		1.7 (1.6)		1.4 (1.5)		4.0 (3.6)	
Professional	6.3 (5.2)		3.0 (3.6)		3.7 (2.8)		5.6 (4.5)		12.2 (10.5)	
Clerk	7.5 (5.4)		1.6 (1.6)		2.0 (2.1)		4.4 (3.9)		8.0 (6.6)	
Service and Sales	5.8 (5.4)		2.6 (4.2)		2.2 (3.3)		4.4 (5.7)		9.2 (13.0)	
Others ^2^	5.7 (4.1)		2.6 (4.0)		3.0 (3.0)		4.9 (4.9)		10.5 (11.3)	
Unemployed	3.5 (3.5)		2.5 (3.5)		2.0 (1.4)		3.5 (2.1)		8.0 (7.0)	
Family status ^1^		0.032		0.065		0.034		0.182		0.057
Nuclear family	7.3 (5.5)		2.6 (3.2)		2.8 (3.0)		4.6 (4.5)		10.1 (10.1)	
Single family	11.9 (7.4)		5.5 (6.7)		4.9 (4.5)		7.0 (6.3)		17.4 (16.1)	
Extended family	5.2 (4.3)		1.7 (2.3)		1.6 (2.1)		3.3 (3.3)		6.6 (7.1)	
Others	5.3 (5.9)		1.3 (1.5)		1.0 (1.7)		2.3 (2.1)		4.7 (4.5)	
Personal history of atopy		0.261		0.255		0.108		0.170		0.145
Yes	6.4 (4.9)		2.1 (3.5)		2.1 (2.8)		3.8 (4.0)		7.9 (9.5)	
No	7.6 (6.0)		2.9 (3.4)		3.0 (3.1)		4.9 (4.6)		10.8 (10.5)	
Family history of atopy		0.343		0.237		0.004		0.097		0.034
Yes	6.3 (5.1)		1.9 (2.6)		1.6 (1.9)		3.3 (3.4)		6.9 (7.1)	
No	7.3 (5.7)		2.8 (3.7)		3.0 (3.3)		4.8 (4.7)		10.6 (11.1)	
Child’s characteristics										
Age (years)	−0.066	0.342	−0.059	0.414	−0.006	0.934	−0.075	0.286	−0.064	0.351
Gender		0.884		0.819		0.556		0.642		0.765
Male	7.0 (5.4)		2.5 (2.8)		2.7 (2.9)		4.5 (3.9)		9.7 (8.9)	
Female	6.9 (5.6)		2.6 (4.4)		2.3 (3.2)		4.1 (5.1)		9.0(12.1)	
Age at eczema onset (months)	−0.094	0.196	−0.026	0.733	−0.026	0.728	−0.059	0.424	−0.046	0.524
Eczema severity by POEM	0.367	<0.001	0.080	0.406	0.194	0.042	0.120	0.213	0.137	0.154
General outpatient visits due to eczema exacerbations over the past 12 months		0.009		0.573		0.520		0.857		0.939
None	6.2 (5.1)		2.6 (3.6)		2.5 (2.9)		4.4 (4.5)		9.5 (10.4)	
≥1	9.4 (6.1)		2.2 (3.0)		2.9 (3.4)		4.2 (3.9)		9.3 (9.5)	
Private practitioner’s clinic visits due to eczema exacerbations over the past 12 months		0.761		0.120		0.940		0.306		0.320
None	6.9 (5.7)		2.8 (3.6)		2.6 (3.1)		4.6 (4.6)		9.9 (10.7)	
≥1	7.3 (5.1)		1.5 (2.5)		2.5 (2.8)		3.5 (3.2)		7.6 (7.5)	
Emergency care visits due to eczema exacerbations over the past 12 months, median (interquartile range)		0.370		0.256		0.851		0.658		0.987
None	5 (3–10)		1 (0–4)		2 (0–3)		3 (1–7)		6 (2–13)	
≥1	11 (2.8–15.5)		0 (0–3.8)		1.5 (0–6)		4.5 (1.8–6.5)		6 (1.8–16.3)	
Hospitalization due to eczema exacerbations over the past 12 months, median (interquartile range)		0.370		0.256		0.851		0.658		0.987
None	5 (3–10)		1 (0–4)		2 (0–3)		3 (1–7)		6 (2–13)	
≥1	11 (2.8–15.5)		0 (0–3.8)		1.5 (0–6)		4.5 (1.8–6.5)		6 (1.8–16.3)	

Note. DASS-21 = The 21-item Depression Anxiety Stress Scale-21; PIQoL-AD = The 28-item Parent’s Index of Quality of Life in Atopic Dermatitis; POEM = Patient-Oriented Eczema Measure; *SD* = standard deviation; *p* = *p*-value; *r* = Pearson correlation coefficient. ^1^ Missing value < 5%. ^2^ Other job occupations include security guards, civil servants, nurses, clinic assistants, and freelancers.
